# Counterregulatory response to hypoglycemia during a hypoglycemic clamp in people with type 2 diabetes treated with tirzepatide

**DOI:** 10.3389/fendo.2025.1627947

**Published:** 2025-09-02

**Authors:** Thomas R. Pieber, Eva Svehlikova, Shweta Urva, Axel Haupt, Chunmei Zhou, Tamer Coskun, Vera Höller, Gabriele Fluhr, Chrisanthi A. Karanikas, Zvonko Milicevic, Edward John Pratt

**Affiliations:** ^1^ Division of Endocrinology and Diabetology, Department of Internal Medicine, Medical University of Graz, Graz, Austria; ^2^ Eli Lilly and Company, Indianapolis, IN, United States; ^3^ Eli Lilly and Company, Vienna, Austria; ^4^ Lilly Centre for Clinical Pharmacology Pte Ltd, Singapore, Singapore

**Keywords:** type 2 diabetes, tirzepatide, counterregulatory response, hypoglycemia, clinical trial

## Abstract

**Introduction:**

To evaluate counterregulatory hormonal responses during a hypoglycemic clamp with tirzepatide.

**Methods:**

Participants with type 2 diabetes (N=42) were randomized to tirzepatide (15 mg) or placebo for 12 weeks in a crossover design, with a wash-out period of 8–10 weeks. The primary objective was the change in glucagon response during clamp-induced hypoglycemia from plasma glucose (PG) 100 mg/dL to nadir PG (45 mg/dL). Secondary measures were changes in responses of other counterregulatory hormones during clamp-induced hypoglycemia. Time to recovery from the nadir to 72 mg/dL and hypoglycemic symptom scores using the 7-point Edinburgh Hypoglycemia Symptom scale were also assessed.

**Results:**

At 12 weeks, HbA1c change from baseline was -1.5% with tirzepatide versus +0.5% with placebo. During induced hypoglycemia, mean PG levels at nadir were 44.5 mg/dL with tirzepatide and 47.5 mg/dL with placebo. Increases in glucagon from PG 100 mg/dL to nadir PG and during recovery from the nadir to 72 mg/dL did not differ between treatments (p=0.756 and p=0.565, respectively). Growth hormone and adrenaline responses did not differ between treatments. Cortisol and noradrenaline responses were delayed with tirzepatide, consistent with lower hypoglycemic symptom scores at nadir observed during tirzepatide treatment periods versus placebo (p=0.007). The proportion of participants aware of hypoglycemia did not differ between treatments.

**Discussion:**

The response of the key counterregulatory hormone glucagon to induced hypoglycemia was maintained with tirzepatide.

**Clinical trial registration:**

ClinicalTrials.gov, identifier NCT04050553.

## Introduction

With its potential to disrupt cerebral function, hypoglycemia is increasingly recognized as an important cause of impaired quality of life, morbidity, and mortality in people with type 2 diabetes (T2D) (1–4). Management of T2D involves a delicate balance of maintaining glycemic control while minimizing hypoglycemic risk (5). However, many glucose-lowering medications, particularly those that increase circulating insulin in a glucose-independent manner, can cause hypoglycemia (6).

Treatment with glucagon-like peptide-1 receptor agonists (GLP-1RAs) is associated with low hypoglycemia risk (7) due to enhancement of insulin secretion that is glucose-dependent (8) and preserved glucagon response to hypoglycemia (9, 10). Glucose-dependent insulinotropic polypeptide (GIP) dose-dependently increases glucagon secretion under euglycemic and hypoglycemic conditions in healthy individuals (11, 12) and in people with type 1 diabetes (T1D) and T2D (12–14), with no direct effect on insulin secretion in healthy individuals (11, 12). Conversely, under hyperglycemic conditions in healthy individuals, GIP has no effect on glucagon responses while strongly enhancing insulin secretion (12).

Tirzepatide is a once weekly dual GIPR/GLP-1R agonist approved for the treatment of T2D, obesity, and obstructive sleep apnea (in the US). In the Phase 3 SURPASS program, superior improvements in glycated hemoglobin (HbA1c) reductions ranging from 1.87-2.59% (20.4-28.3 mmol/mol) and body weight reductions ranging from 6.2-12.9 kg (6.6-13.9%]) were associated with tirzepatide versus placebo and active comparators in participants with T2D on a variety of concomitant therapies (15–19). Improvements in glycemic control were observed without increased risk of clinically significant (blood glucose (BG) <54 mg/dL [3 mmol/L]) or severe hypoglycemia (15–19).

Because of a potential differential effect of GIPR signaling on glucagon secretion, it is important to fully characterize counterregulatory response with tirzepatide. We investigated the response of glucagon and other counterregulatory hormones, insulin and C-peptide concentration, hypoglycemic symptoms, and cognitive function during clamp-induced hypoglycemia in people with T2D treated with tirzepatide versus placebo.

## Materials and methods

### Trial design and participants

**Figure 1:**
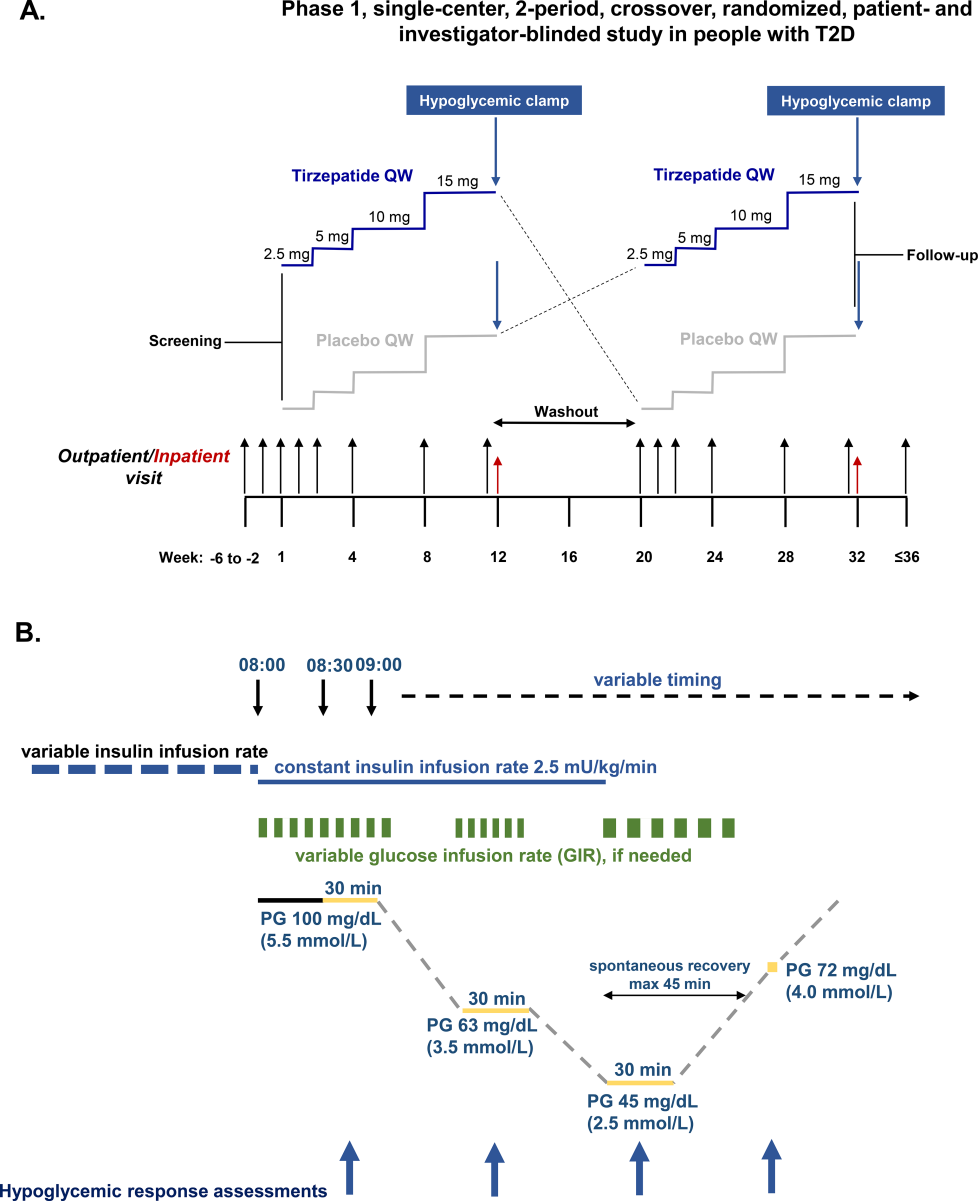
Study design and hypoglycemic clamp procedure. **(A)** Study design. **(B)** Hypoglycemic clamp procedure. Glucagon, fasting insulin, C-peptide, cortisol, growth hormone, adrenaline, and noradrenaline measured at 10, 20 and 30 min of each clamp plateau and once PG 72 mg/dL (4.0 mmol/L) was reached. Blood pressure and pulse rate measured at 0 and 30 minutes of each plateau and once PG 72 mg/dL (4.0 mmol/L) was reached. Hypoglycemic symptom score and hypoglycemic symptom awareness assessed at 0 and 30 min of each plateau and once PG 72 mg/dL (4.0 mmol/L) was reached. PG, plasma glucose; QW, once weekly.

This Phase 1, single-center, two-period, crossover, randomized, double-blind study, conducted in Austria (Clinical Trials Unit, Medical University of Graz, Graz, Austria), assessed the secretion of counterregulatory hormones in response to a hypoglycemic stimulus and parameters of recovery from the nadir glucose levels to normoglycemia defined by plasma glucose (PG) 72 mg/dL (4.0 mmol/L), in tirzepatide-treated participants versus placebo. Following lead-in, eligible participants were randomized (1:1) to 12 weeks of tirzepatide (15 mg) followed by 12 weeks of placebo or vice versa (**Figure 1A**). A washout period of 8–10 weeks between the final dose of study drug in the first treatment period (on Week 12) and the first dose of study drug in the second treatment period was implemented based on the elimination half-life of tirzepatide (20). Key eligibility criteria included adults aged 18–70 years with T2D (HbA1c [6.5-9.0% [46–75 mmol/mol] at screening if taking metformin only or an HbA1c 6.0-8.5% [42–69 mmol/mol] at screening if taking metformin plus an additional oral antidiabetic medication [OAM]), and a body mass index (BMI) of 23.0-45.0 kg/m^2^. Participants continued metformin treatment and those treated with any other OAM underwent an investigator-monitored washout of these additional drugs during a 4-week lead-in prior to randomization. A full list of eligibility criteria is in the **Supplementary Appendix**. The tirzepatide treatment period included stepwise dose escalation (**Supplementary Table S1**).

This trial was conducted in accordance with the International Conference on Harmonisation Guidelines for Good Clinical Practice and the Declaration of Helsinki. All participants provided signed informed consent, and protocols were approved by local ethical review boards.

### Hypoglycemic clamp procedure

Key timepoints for the hypoglycemic clamp and a schematic are in **Supplementary Table S2**; **Figure 1B**. The last treatment dose for each period was given on Day 78 (± 2 days). The clamp procedure was initiated on Day 79 (clamp run-in period) and hypoglycemic induction was performed on Day 80 of each period using constant insulin i.v. infusion 2.5 mU/kg/min. The target PG level of 100 mg/dL (5.5 mmol/L) was maintained for 60 min before hypoglycemia induction, when PG was allowed to decline to 63 mg/dL (3.5 mmol/L) and subsequently to nadir (target PG 45 mg/dL [2.5 mmol/L]). At each PG level, a variable glucose infusion rate was used to maintain the target stable for 30 min and assessments of the hypoglycemic response were made, including blood sampling. PG <45 mg/dL was not permitted. If the target could not be reached within 30 min, a higher PG for the target plateau was acceptable for safety reasons. Once reached, the constant insulin infusion was terminated and the variable glucose restarted, if needed, to maintain the target nadir plateau for 30 min. The glucose infusion was then switched off to allow spontaneous recovery from the nadir to PG 72 mg/dL. If the PG had not reached ≥72 mg/dL 45 min after termination of the insulin infusion, PG was raised to 72 mg/dL using a constant glucose infusion. After recovery assessments were performed, PG was actively raised to 100 mg/dL by variable glucose infusion.

If hypoglycemia symptoms became intolerable during the hypoglycemic clamp procedure, glucose was infused to increase PG and PG concentrations above 45 mg/dL would be considered as the nadir.

### Endpoints and assessments during the hypoglycemic clamp

The primary objective was assessment of glucagon response during induced hypoglycemia from a target PG 100 mg/dL to a nadir 45 mg/dL in people with T2D following 12 weeks of tirzepatide 15 mg versus placebo. Key secondary objectives included change in glucagon response from a target PG 100 to 63 mg/dL and to recovery from the nadir (PG 72 mg/dL), changes in glucagon response from ambient PG (measured in the pre-prandial state prior to clamp initiation) to target PG 100, 63, 45 mg/dL, and to recovery from the nadir (PG 72 mg/dL). Additional secondary objectives included assessments of changes in mean adrenaline, noradrenaline, cortisol, growth hormone (GH), insulin, and C-peptide concentrations from a target PG 100, 63, and 45 mg/dL, and to recovery from the nadir (PG 72 mg/dL), and the area under the curve for glucose infusion rate (AUC_GIR_) at each PG target. Symptoms of hypoglycemia questionnaires were applied at 0 min and 30 min of each PG plateau and at recovery from the nadir, using the 7-point Edinburgh Hypoglycemia Symptom scale (21). Thirteen questions were used to measure neuroglycopenic symptoms (i.e., cognitive dysfunction including inability to concentrate, confusion, blurred vision, difficulty speaking, anxiety, and double vision, and neuroglycopenia including drowsiness, tiredness, hunger, and weakness) and autonomic symptoms (i.e., sweating, trembling and warmness). This is a subjective, validated questionnaire that measures the intensity of commonly experienced hypoglycemic symptoms where 1 represents no symptoms and 7 represents severe symptoms (i.e., a lower score indicates less severe symptoms). Sub-scores for neuroglycopenic and autonomic symptoms and overall scores were calculated. Hypoglycemic awareness was evaluated at 0 min and 30 min of each PG plateau and at recovery from the nadir based on the participant’s response to the question “Do you feel hypoglycemic” (yes/no).

### Laboratory assessments

Blood samples were collected for in-protocol biomarkers including glucagon, insulin and C-peptide, GH, cortisol, adrenaline, and noradrenaline at fasting state and during the hypoglycemic plateaus, were measured at a central laboratory (Labcorp Central Laboratory Services, Geneva, Switzerland). PG concentrations during hypoglycemic clamp were analyzed using SUPER GL compact (Dr. Müller Gerätebau GmbH, Freital, Germany).

### Safety parameters

Safety assessments, including evaluation of adverse events (AEs), clinical laboratory parameters, vital signs, electrocardiograms, injection site and hypersensitivity reactions, were performed throughout the hypoglycemic clamps and pharmacodynamic assessments were performed during each plateau. Outside of the hypoglycemic clamp, PG levels were regularly monitored at a minimum of once daily (fasting PG) or in case of any hypoglycemic symptoms or required by participant. Hypoglycemic events are defined in the **Supplementary Appendix**.

### Statistical analyses

A sample size of 30 completers was determined based on the criterion that the width of the 95% confidence interval (CI) for the treatment comparison of the primary endpoint was within ±23.65 pg/mL with ~80% probability, provided that within-subject variability was 40 pg/mL (10).

Safety analyses were conducted on all randomized participants exposed to ≥1 dose of study drug (safety population), and pharmacodynamic analyses were conducted on evaluable pharmacodynamic data from all exposed participants who complete both hypoglycemic clamp procedures (pharmacodynamic analysis set). All tests of treatment effects were conducted at a 2-sided alpha=0.05.

The primary pharmacodynamic analysis assessed the treatment difference on the change in mean glucagon concentration from the target PG plateau of 100 mg/dL to the nadir target PG plateau of 45 mg/dL, using a linear mixed-effects model with treatment, treatment period, and treatment sequence as fixed effects and participants as a random effect, using a restricted maximum likelihood method. Statistical analyses for secondary continuous pharmacodynamic variables were conducted in a similar manner. A nonparametric McNemar test at each target PG plateau and at recovery from the nadir was used to assess differences in percent of participants treated with tirzepatide versus placebo who were aware of hypoglycemia.

Sensitivity analyses were conducted for the primary endpoint using only data from those participants who achieved the target PG nadir plateau of 45 mg/dL.

## Results

### Trial population and baseline characteristics

This trial was conducted between February 25, 2020 and January 25, 2022. Forty-two randomized participants received ≥1 dose of study drug (**Supplementary Figure S1**). Nine participants did not complete the trial due to the COVID-19 pandemic (tirzepatide: n=2; placebo: n=2), withdrawal by participant (tirzepatide: n=2), and AEs (tirzepatide: n=2, placebo: n=1). Participants in the pharmacodynamic population (N=33) had a mean baseline age of 57.5 years, 84.8% were male with a mean duration of diabetes of 8.2 years, HbA1c 7.5% (58.8 mmol/mol), and BMI 30.9 kg/m^2^ (**Table 1**). Similar ranges were observed in the safety population (N=42).

**Table 1:** Baseline demographics and clinical characteristics.

Parameter	Total N=33
Age, years	57.5 ± 5.5
Female, n (%)	5 (15.2)
Race, n (%)	
White	33 (100.0)
Ethnicity, n (%)	
Not Hispanic or Latino	33 (100.0)
Duration of diabetes, years	8.2 ± 4.7
HbA1c, mmol/mol	58.8 ± 9.0
HbA1c, %	7.5 ± 0.8
Fasting glucagon, pmol/L	12.3 ± 4.5
Fasting insulin, pmol/L	76.5 ± 42.0
Fasting C-peptide, pmol/L	799.3 ± 295.0
Fasting glucose, mmol/L	9.2 ± 2.1
Weight, kg	94.7 ± 14.4
Body mass index, kg/m^2^	30.9 ± 4.1
Systolic blood pressure, mmHg	135.8 ± 13.7
Diastolic blood pressure, mmHg	85.1 ± 7.9
Pulse rate, bpm	69.2 ± 8.3

Data are mean ± SD, unless otherwise specified. Pharmacodynamic population. Bpm, beats per minute; HbA1c, glycated hemoglobin; SD, standard error.

### Glucagon response during clamp-induced hypoglycemia and recovery from the nadir to 4.0 mmol/L

**Figure 2:**
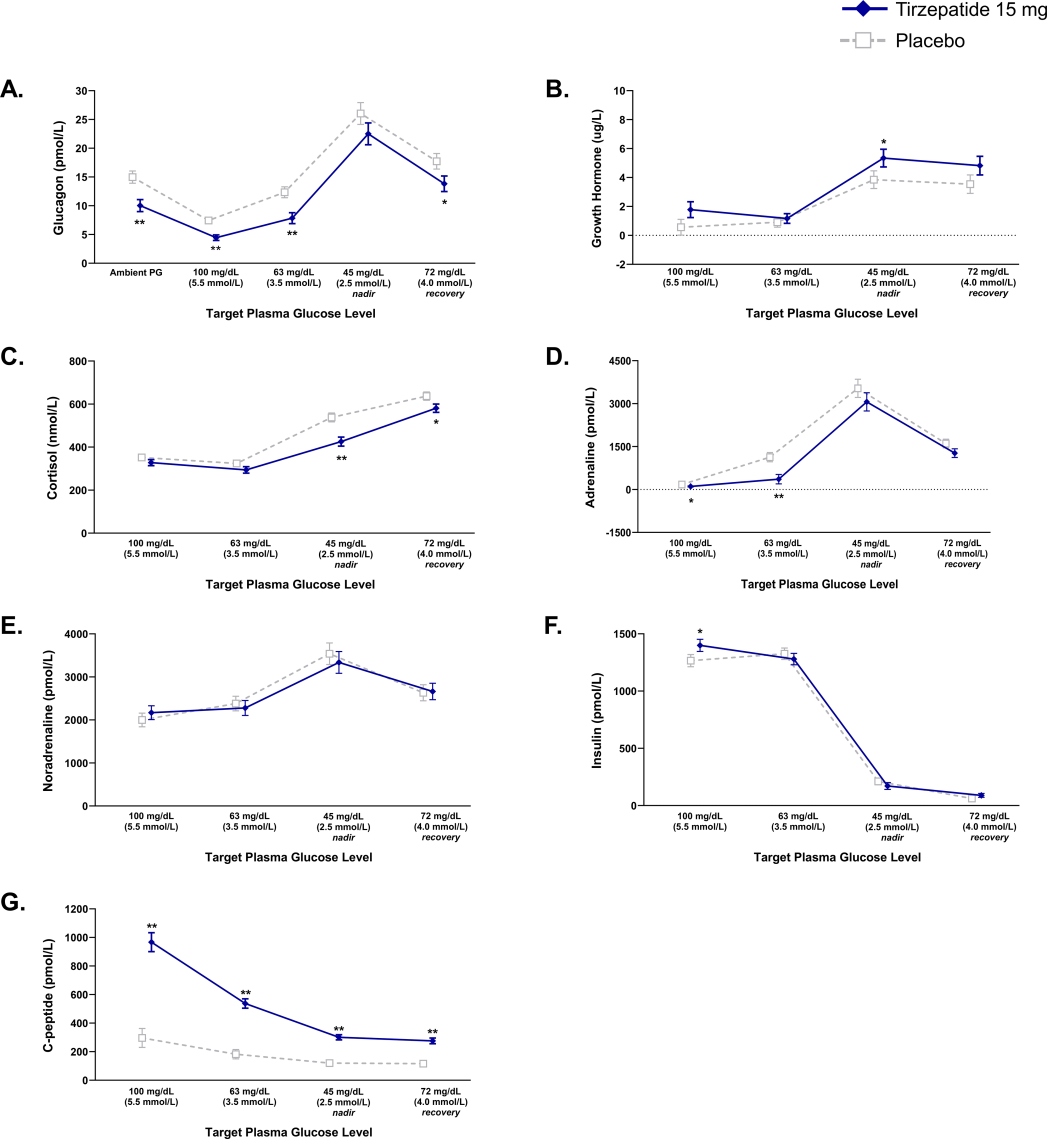
Responses of counterregulatory hormones, insulin, and C-peptide during hypoglycemic clamp. Data are LSM (SE). Pharmacodynamic population. **(A)** Glucagon. **(B)** Growth Hormone. **(C)** Cortisol. **(D)** Adrenaline. **(E)** Noradrenaline. **(F)** Insulin. **(G)** C-peptide. *p<0.05 and **p<0.001 versus placebo. LSM, least squares mean; PG, plasma glucose; SE, standard error.

Absolute glucagon concentrations were significantly lower with tirzepatide versus placebo at ambient glucose concentrations and all target PG plateaus, except PG 45 mg/dL (**Figure 2A**). From mean values of 4.48 pmol/L in the tirzepatide group and 7.45 pmol/L in the placebo group at a target PG 100 mg/dL, the primary analysis showed that increases in mean glucagon concentration in response to induced hypoglycemia from the target PG 100 mg/dL to nadir did not differ between treatments (**Table 2**). Sensitivity analyses including only those participants who reached the target PG 45 mg/dL (tirzepatide: N=30, placebo: N=22) showed similar results (18.15 versus 20.98 pmol/L; treatment difference [95% CI], -2.83 pmol/L [-6.45, 0.80], p=0.120).

**Table 2:** Analysis of counterregulatory hormone responses, and responses of insulin and C-peptide during induced hypoglycemia.

Parameter	Actual Value at PG 5.5 mmol/L (Tirzepatide 15 mg/Placebo)	Change from PG 100 mg/dL (5.5 mmol/) to Target	Tirzepatide 15 mg (N=32-33)	Placebo (N=32-33)	Estimated Treatment Difference (95% CI)	P-value
Glucagon, pmol/L	4.48 (2.88)/7.45 (2.69)	PG 63 mg/dL	3.40 (0.65)	4.90 (0.65)	-1.50 (-3.26, 0.27)	0.094
		PG nadir 45 mg/dL	18.07 (1.62)	18.55 (1.64)	-0.49 (-3.64, 2.67)	0.756
		Recovery PG 72 mg/dL	9.38 (1.14)	10.27 (1.14)	-0.89 (-4.02, 2.24)	0.565
Growth Hormone, ug/L	1.74 (4.40)/0.57 (0.63)	PG 63 mg/dL	-0.62 (0.47)	0.34 (0.47)	-0.96 (-2.20, 0.29)	0.128
		PG nadir 45 mg/dL	3.58 (0.88)	3.31 (0.88)	0.27 (-1.88, 2.42)	0.799
		Recovery PG 72 mg/dL	3.05 (0.93)	2.98 (0.91)	0.07 (-2.13, 2.27)	0.947
Cortisol, nmol/L	327.64 (70.66)/353.09 (88.01)	PG 63 mg/dL	-33.36 (11.53)	-28.08 (11.37)	-5.29 (-35.13, 24.55)	0.720
		PG nadir 45 mg/dL	99.00 (20.48)	187.40 (20.48)	-88.40 (-141.73, -35.07)	0.002
		Recovery PG 72 mg/dL	253.12 (20.88)	284.83 (20.66)	-31.71 (-75.65, 12.22)	0.151
Adrenaline, pmol/L	111.13 (98.14)/177.53 (121.27)	PG 63 mg/dL	254.77 (156.26)	955.38 (153.31)	-700.61 (-1089.34, -311.87)	0.001
		PG nadir 45 mg/dL	2957.63 (317.08)	3361.95 (315.92)	-404.32 (-960.75, 152.10)	0.148
		Recovery PG 72 mg/dL	1165.56 (149.15)	1431.43 (146.30)	-265.87 (-643.27, 111.53)	0.161
Noradrenaline, pmol/L	2171.19 (1002.76)/2012.68 (805.99)	PG 63 mg/dL	110.01 (69.26)	383.50 (67.93)	-273.49 (-449.81, -97.17)	0.004
		PG nadir 45 mg/dL	1173.53 (180.49)	1537.20 (179.91)	-363.67 (-657.75, -69.60)	0.017
		Recovery PG 72 mg/dL	497.23 (101.71)	632.33 (100.31)	-135.10 (-331.39, 61.20)	0.170
Insulin, pmol/L	1401.68 (363.88)/1269.56 (288.80)	PG 63 mg/dL	-119.55 (23.37)	60.83 (23.37)	-180.38 (-242.74, -118.02)	<0.001
		PG nadir 45 mg/dL	-1228.08 (53.61)	-1053.31 (54.20)	-174.77 (-290.34, -59.21)	0.004
		Recovery PG 72 mg/dL	-1315.89 (54.73)	-1202.03 (54.99)	-113.86 (-227.81, 0.09)	0.050
C-peptide, pmol/L	966.60 (506.90)/297.80 (154.00)	PG 63 mg/dL	-429.44 (36.84)	-114.16 (36.84)	-315.27 (-407.45, -223.10)	<0.001
		PG nadir 45 mg/dL	-665.99 (51.09)	-177.69 (51.78)	-488.31 (-621.25, -355.36)	<0.001
		Recovery PG 72 mg/dL	-690.96 (52.78)	-180.06 (52.78)	-510.89 (-654.01, -367.78)	<0.001

Data are mean (SD) actual value at PG 100 mg/dL (5.5 mmol/L), LSM (SE) change from PG 100 mg/dL (5.5 mmol/L) to target PG 63 mg/dL (3.5 mmol/L), PG nadir 45 mg/dL (2.5 mmol/L), and recovery from the nadir to PG 72 mg/dL (4.0 mmol/L) and estimated treatment difference (95% CI) at Week 12. CI, confidence interval; LSM, least squares mean; PG, plasma glucose; SD, standard deviation; SE, standard error.

Secondary analysis showed that increases in mean glucagon concentration with tirzepatide versus placebo did not differ in response to induced hypoglycemia from the target PG 100 mg/dL to PG 63 mg/dL and to recovery from the nadir (PG 72 mg/dL) (**Table 2**).

### Other counterregulatory hormone responses during clamp-induced hypoglycemia and recovery from the nadir (PG 72 mg/dL)

Absolute GH concentrations were higher with tirzepatide versus placebo at target PG 45 mg/dL (**Figure 2B**). From mean values of 1.74 ug/L in the tirzepatide group and 0.57 ug/L in the placebo group at target PG 100 mg/dL, changes in GH did not differ versus placebo at any other PG target (**Table 2**). Absolute cortisol concentrations were lower with tirzepatide versus placebo at nadir and at recovery from the nadir (PG 72 mg/dL) (**Figure 2C**). From mean values of 328 nmol/L in the tirzepatide group and 353 nmol/L in the placebo group at target PG 100 mg/dL, increases in cortisol to nadir were smaller with tirzepatide versus placebo (**Table 2**). Absolute adrenaline concentrations were lower with tirzepatide versus placebo at target PG 100 mg/dL and at target PG 63 mg/dL (**Figure 2D**). From mean values of 111 pmol/L in the tirzepatide group and 178 pmol/L in the placebo group at target PG 100 mg/dL, increases in adrenaline to target PG 63 mg/dL were smaller with tirzepatide versus placebo. The changes in mean adrenaline concentrations at target PG nadir of 45 mg/dL and recovery from the nadir did not differ when receiving tirzepatide versus placebo (**Table 2**). Absolute noradrenaline concentrations did not differ between treatments at any target PG plateau (**Figure 2E**). From mean values of 2171 pmol/L in the tirzepatide group and 2013 pmol/L in the placebo group at target PG 100 mg/dL, increases in noradrenaline to target PG 63 mg/dL and to 45 mg/dL were smaller with tirzepatide versus placebo, whereas changes in mean noradrenaline concentrations from PG 100 mg/dL to recovery from the nadir did not differ between treatments (**Table 2**).

### Insulin and C-peptide response during clamp-induced hypoglycemia and recovery from the nadir (PG 72 mg/dL)

Mean absolute insulin concentration at the PG 100 mg/dL was higher with tirzepatide versus placebo (**Figure 2F**). From mean values of 1402 pmol/L in the tirzepatide group and 1270 pmol/L in the placebo group at target PG 100 mg/dL, decreases in insulin from a target PG 100 mg/dL to a target PG 63 mg/dL and to nadir were greater with tirzepatide versus placebo (**Table 2**). Mean absolute C-peptide concentrations at all target PG plateaus were higher with tirzepatide versus placebo (**Figure 2G**). From mean values of 967 pmol/L in the tirzepatide group and 298 pmol/L in the placebo group at target PG 100 mg/dL, decreases in C-peptide from a target PG 100 mg/dL to all target PG plateaus were greater with tirzepatide versus placebo (**Table 2**).

### Time to reach recovery from the nadir to 72 mg/dL

Mean time to recovery from the nadir to 72 mg/dL was 47.7 min with tirzepatide and 43.3 min with placebo (estimated treatment difference [ETD] [95% CI]: 4.39 min [1.69, 7.08]; p=0.002). However, nadir PG was lower with tirzepatide (2.47 versus 2.63 mmol/L; p=0.015). Additional sensitivity analysis showed that when the nadir PG achieved was considered, the significant difference in the time taken to recover from hypoglycemia when receiving tirzepatide versus placebo was reduced (47 versus 44 min; ETD: 3.09 min [0.02, 6.17]; p=0.049).

### Glucose infusion rate

The AUC_GIR_ was higher with tirzepatide during each of the PG plateaus of the hypoglycemic induction versus placebo, indicating improvement in insulin sensitivity (**Supplementary Figure S2**).

### Hypoglycemic symptoms and cognitive function tests

**Figure 3:**
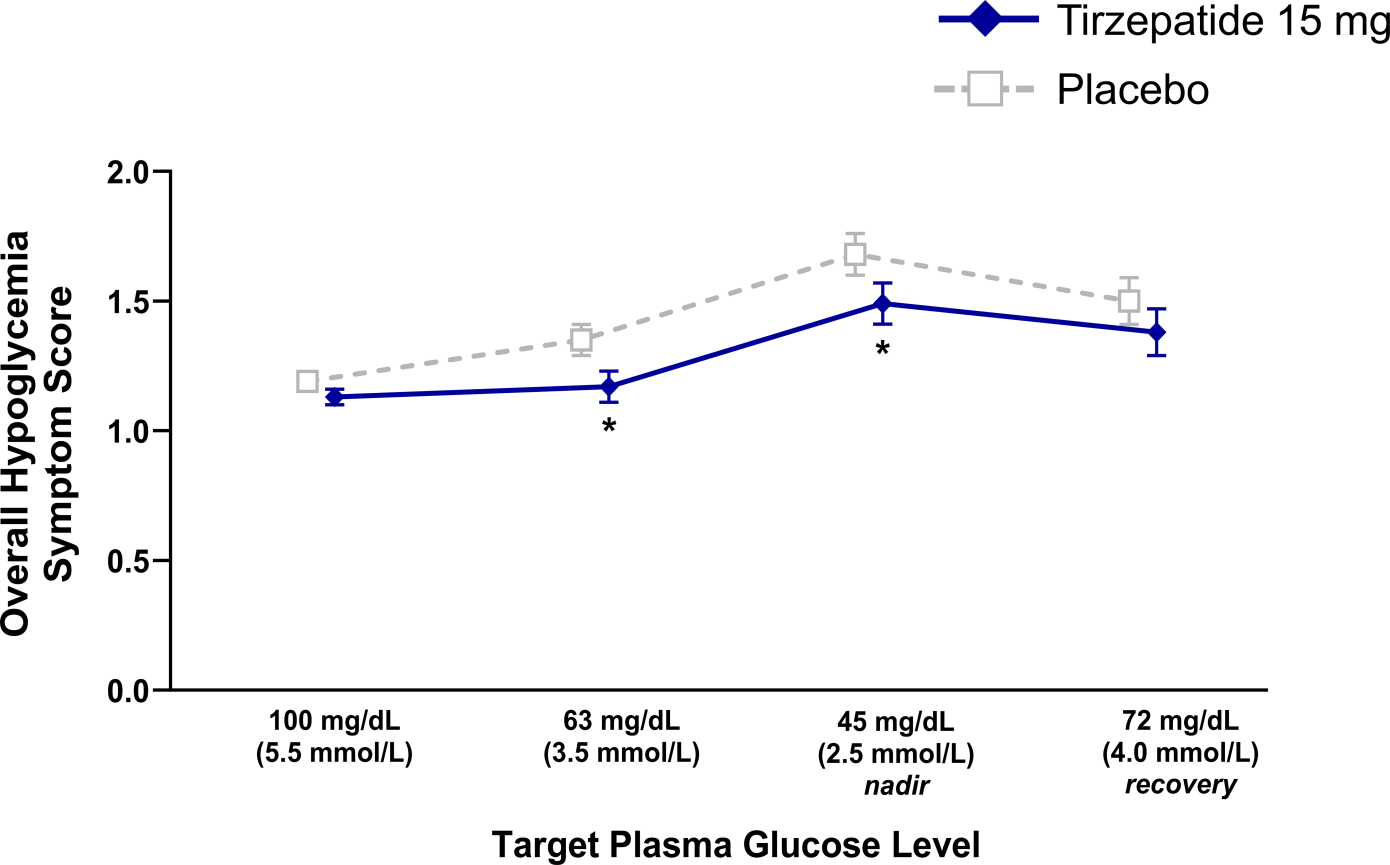
Overall hypoglycemic symptom score data during induced hypoglycemia. Data are LSM (SE). Pharmacodynamic population. Lower scores indicate fewer symptoms. *p<0.05 versus placebo. LSM, least squares mean; SE, standard error.

Mean overall hypoglycemic symptom scores at the target PG plateaus of 63 and 45 mg/dL were 1.17 and 1.49 with tirzepatide and 1.35 and 1.68 with placebo, respectively (**Figure 3**, **Supplementary Table S3**). Mean overall hypoglycemic symptom scores were lower with tirzepatide versus placebo (ETD: at target PG 63 mg/dL: -0.18 [-0.31, -0.05]; p=0.010; ETD at target PG 45 mg/dL: -0.19 [-0.32, -0.06]; p=0.007). Cognitive dysfunction symptoms were significantly lower with tirzepatide at target PG 63 mg/dL (ETD -0.11 [-0.21, 0.00]; p=0.046) (**Supplementary Table S3**), which was mainly driven by higher scores for the symptom “inability to concentrate” in the placebo group (**Supplementary Table S4**). Neuroglycopenia symptoms were significantly lower with tirzepatide at targets PG 63 mg/dL and PG 45 mg/dL (ETD -0.31 [-0.56, -0.06] p=0.017 and -0.28 [-0.52, -0.04]; p=0.022, respectively) (**Supplementary Table S3**), which was mainly driven by higher scores for the symptom “drowsiness” and “hunger” in the placebo group (**Supplementary Table S4**). Finally, autonomic symptoms were significantly lower with tirzepatide at target PG 45 mg/dL (ETD -0.36 [-0.60, -0.12]; p=0.005) (**Supplementary Table S3**), which was mainly driven by higher scores for the symptom “sweating” and “warmness” in the placebo group (**Supplementary Table S4**). Symptoms were transient and were not associated with any adverse events.

The proportions of participants aware of hypoglycemia during induced hypoglycemia and recovery from the nadir (PG 72 mg/dL) were similar between treatments (**Supplementary Figure S3**). All tirzepatide-treated participants answered “no” to the question “Do you feel hypoglycemic?” at the target levels of 100 mg/dL, whereas 3 (9.1%) placebo-treated participants answered “yes”.

### Changes in HbA1c and body weight

At Week 12, mean HbA1c change from baseline was -1.5% (-16.4 mmol/L) with tirzepatide versus +0.5% (+5.8 mmol/L) with placebo. Mean body weight changes from baseline were -7.6 kg versus +1.3 kg, respectively.

### Tirzepatide pharmacokinetics

Geometric mean tirzepatide concentrations ranged from 296.4 ng/mL at the end of the second week of dosing with tirzepatide 5 mg to 947.3 ng/mL at the end of the third week of dosing with tirzepatide 15 mg.

### Safety

No deaths were reported, and four participants reported single serious adverse events (SAEs) during tirzepatide treatment (see **Supplementary Table S5** for details on individual cases of SAEs and study discontinuation due to AEs).

The incidence of treatment-emergent AEs related to study drug was higher with tirzepatide versus placebo, and mostly gastrointestinal in nature (nausea, vomiting, diarrhea) and decreased appetite (**Supplementary Table S5**).

No severe or clinically significant hypoglycemic events occurred outside of the hypoglycemic clamp. Nine hypoglycemic events were reported with tirzepatide (15 mg, n=7; 10 mg, n=2). Eight of these events were documented asymptomatic hypoglycemia with a PG ≤70 mg/dL (3.9 mmol/L) but ≥54 mg/dL and one was symptomatic with a PG >70 mg/dL. One placebo-treated participant reported one documented symptomatic hypoglycemic event with a PG 67 mg/dL (3.7 mmol/L). All participants promptly recovered, the majority following food intake.

Mean changes in clinical laboratory values and safety vital signs were similar between the treatment periods. Pulse rate (PR) was higher at all PG plateaus by up to 11 bpm, systolic blood pressure (BP) at the target PG 100 mg/dL and 45 mg/dL was lower by up to 8 mmHg, and diastolic BP at the target PG 100 mg/dL was lower by ~4 mmHg with tirzepatide versus placebo (**Supplementary Figure S4**). Changes in PR and BP in response to induced hypoglycemia and recovery from the nadir (PG 72 mg/dL) did not differ between treatments.

## Discussion

Hypoglycemia activates a counterregulatory hormonal cascade to rapidly restore euglycemia. Herein, counterregulatory hormonal response was assessed in people with T2D treated with tirzepatide 15 mg versus placebo during a hypoglycemic clamp procedure. Glucagon response to low BG levels was maintained with tirzepatide similar to that observed with placebo. GH response was also similar during both treatment periods, while cortisol response was delayed. Catecholamine responses were variable prior to the 45 mg/dL nadir, with no difference in adrenaline at nadir and during recovery from the nadir. Noradrenaline response was smaller at nadir with tirzepatide but did not differ during recovery from the nadir. Participants reported less intensive hypoglycemic symptoms with tirzepatide with no difference between treatments in ability to detect hypoglycemia at nadir. Overall, the response to hypoglycemia was not affected by tirzepatide, consistent with observed data on hypoglycemic risk in the Phase 3 SURPASS clinical trial program for T2D (15–19).

Hypoglycemia is an important clinical event associated with multiple negative consequences, ranging from disruption of daily activities to a direct increase in potentially fatal cardiovascular events (22). Under hypoglycemic conditions in both healthy individuals and in people with T1D and T2D, native GLP-1 and GLP-1RAs do not exert any effect on glucagon secretion (10, 23–28). In contrast, it has been reported that GIP increases glucagon secretion under euglycemic and hypoglycemic conditions, where it has little or no effect on insulin secretion in healthy individuals (11, 12) and in people with T2D (14). The present study evaluated changes in counterregulatory hormone responses with tirzepatide under hypoglycemic conditions in participants with T2D on concomitant metformin. Standard hypoglycemic clamp methodology was used to induce hypoglycemia and measured parameters of interest at each predefined threshold, including PG levels of 100, 63 and 45 mg/dL, and during recovery from hypoglycemia (PG 72 mg/dL) (29). Glucagon response was chosen as the primary objective because this hormone is the main mediator of the early response to hypoglycemia through increase in hepatic glucose production (via stimulation of gluconeogenesis and glycogenolysis) (30, 31).

Despite lower glucagon levels at the start of the clamp due to the known effect of incretins under euglycemic and hyperglycemic conditions, glucagon response during induced hypoglycemia was maintained with tirzepatide. GH response did not differ, adrenaline response was generally similar between treatments, whereas noradrenaline responses were generally delayed with tirzepatide. The clinical relevance of the delay in cortisol response to acute hypoglycemia may be limited because of glucagon and potentially the effect of catecholamines, albeit to a minor extent. Catecholamines are dominant in the early periods of hypoglycemia, while cortisol and GH significantly contribute only hours after the occurrence of low BG (32–34). Importantly, hormonal responses with tirzepatide were very similar to those reported for selective GLP-1RA, semaglutide (10).

Although minor and of little clinical relevance, participants required more time to reach PG 72 mg/dL with tirzepatide, a pre-specified threshold for recovery from hypoglycemia. The time to reach PG 72 mg/dL may have been confounded by factors known to influence glucose levels that significantly differed between the treatment periods, including lower glucose level at nadir with tirzepatide and strong insulin sensitization associated with tirzepatide (35). The presumed impact of the differences in insulin sensitivity herein is supported by more glucose needed with tirzepatide during the hypoglycemic clamp. The latter effect was reflected in higher glucose infusion rates needed to achieve and keep glucose threshold during the tirzepatide period. Glucose differences at nadir were adjusted, which confirmed its impact on time to recovery from the nadir. We could not adjust for other potential confounders to the data set.

Overall mean hypoglycemia symptom scores were lower at target PG 63 and 45 mg/dL with tirzepatide, indicating a lowering of threshold due to the improved glycemic control observed. This was consistent with other studies showing a normalization (i.e., a decrease) in symptom thresholds with improved glycemic control (10, 36) and may explain some of the differences in counterregulatory hormone responses observed with tirzepatide, such as the delayed increases in cortisol and noradrenaline. Overall hypoglycemic symptom score did not differ between treatments following recovery (PG 72 mg/dL).

Tirzepatide treatment was generally well tolerated in participants with T2D. AEs were mostly gastrointestinal in nature, consistent with other tirzepatide trials (15–19) and with the safety profile of the GLP-1RA class. The safety profile did not differ between treatments.

Study limitations included differences in body weight and glycemic control (as determined by HbA1c and insulin sensitivity) with tirzepatide versus placebo. These differences may affect counterregulatory hormonal responses, including baseline cortisol levels, and improved glycemic control may consequently lower hypoglycemic symptom scores and their threshold, lower insulin infusion rates and increase glucose infusion rates during clamp with tirzepatide, and lower nadir PG with tirzepatide. Additional studies are required to better understand the mechanisms behind the well-preserved glucagon response observed in the current study and to elucidate the potential contribution of GIP agonism in the maintenance of counterregulatory hormonal responses. However, this trial was designed to investigate the response to hypoglycemia during stable tirzepatide treatment, which would be accompanied by improved glycemic control. Therefore, these results may be informative for routine clinical practice. Finally, the current study demonstrated that treatment with tirzepatide did not affect adaptive hormonal responses in the short-term; however, additional studies are required to assess the long-term effects of body weight reduction with tirzepatide treatment on counterregulatory hormonal responses under hypoglycemic conditions. A strength of this study was its crossover design with high power and statistical efficiency, in which all participants underwent both treatment periods, allowing participants to serve as their own control.

In conclusion, the response of the key counterregulatory hormone glucagon to induced hypoglycemia was maintained with tirzepatide in people with T2D. Overall, these results are consistent with those observed in the SURPASS clinical trials, which were associated with low hypoglycemic risk despite robust glycemic control with tirzepatide. These data will help further elucidate the counterregulatory response to a hypoglycemic stimulus with tirzepatide in people with T2D and provide important safety information for potential future clinical use.

## Data Availability

The raw data supporting the conclusions of this article will be made available by the authors, without undue reservation.
