# Supplementary material for: Counterregulatory response to hypoglycemia during a hypoglycemic clamp in people with type 2 diabetes treated with tirzepatide
Source: Front Endocrinol (Lausanne). 2025 Sep 2;16:1627947. doi: 10.3389/fendo.2025.1627947 (PMC12436105; doi:10.3389/fendo.2025.1627947)
Supplement: Supplementary file 1 [file DataSheet1.docx]

**ONLINE-ONLY SUPPLEMENTAL APPENDIX**

**This supplement provides data on:** Pieber TR et al. Counterregulatory Response to Hypoglycemia During a Hypoglycemic Clamp in People with Type 2 Diabetes Treated with Tirzepatide. Front Endocrinol (Lausanne). 2025. *DOI: 10.3389/fendo.2025.1627947*

**CONTENTS**

**SUPPLEMENTAL METHODS**

Inclusion Criteria………………………………………………………………….……………..2

Exclusion Criteria………………………………………………………………….…………….4

Definitions of Hypoglycemia……………………………………………………….…………...6

**SUPPLEMENTAL TABLES**

Table S1. Dose Escalation Schemes………………………………………………………….7

Table S2. Key time points for hypoglycemic clamp…………………………………………..8

Table S3. Hypoglycemic symptom scores during induced hypoglycemia and recovery…9

Table S4. Individual symptom scores during induced hypoglycemia and recovery….…10

Table S5. Safety profile………………………………………………………………………..13

**SUPPLEMENTAL FIGURES**

Figure S1. Participant disposition………………………………………………………..…...14

Figure S2. Glucose infusion-related parameters during induced hypoglycemia……...….15

Figure S3. Hypoglycemia awareness during clamp-induced hypoglycemia……………..16

Figure S4. Vital signs during induced hypoglycemia……………………………………….17

**SUPPLEMENTAL METHODS**

**Inclusion Criteria**

Participant are eligible for inclusion in the study only if they meet all of the following criteria at screening and/or at other visits prior to randomization, when indicated:

**Disease Characteristics**

1. Have had T2DM for at least 1 year;
2. Treated with diet and exercise and a stable dose of metformin (3 months prior to study entry) with or without 1 additional OAM other than metformin; doses of metformin will be considered stable if all prescribed doses for the previous 3 months were ±850 mg from the most commonly prescribed dose; allowed OAMs, in combination with metformin are DPP-IV inhibitors, SGLT-2 inhibitors, acarbose, glinides and sulfonylureas; in participants with established cardiovascular disease who are treated with SGLT-2 inhibitors, this therapy should not be discontinued and therefore, these participants will NOT be eligible for participation in the trial.

3a. Have a hemoglobin A1c value at screening of ≥6.5% and ≤9.0% (48 to 75 mmol/mol), if on

metformin only;

3b. Have a hemoglobin A1c value at screening of ≥6.0% and ≤8.5% (42 to 69 mmol/mol), if on

metformin and 1 more allowed OAM;

**Participant Characteristics**

1. Male or female participants aged 18 to 70 years, inclusive

4a. Male participants:

- Men, regardless of their fertility status, with nonpregnant women of childbearing potential (WOCBP) partners must agree to either remain abstinent (if this was their preferred and usual lifestyle) or use condoms as well as 1 additional highly effective (less than 1% failure rate) method of contraception (such as combination oral contraceptives, implanted contraceptives, or intrauterine devices) or effective method of contraception (such as diaphragms with spermicide or cervical sponges) for the duration of the study and until their plasma concentrations are below the level that could result in a relevant potential exposure to a possible fetus, predicted to be 90 days.
  - Men and their partners may choose to use a double–barrier method of contraception. (Barrier protection methods without concomitant use of a spermicide are not an effective or acceptable method of contraception. Thus, each barrier method must include use of a spermicide. It should be noted, however, that the use of male and female condoms as a double-barrier method was not considered acceptable due to the high failure rate when these barrier methods are combined).
  - Periodic abstinence (e.g., calendar, ovulation, symptothermal, postovulation methods), declaration of abstinence just for the duration of the study, and withdrawal are not acceptable methods of contraception.
- Men with pregnant partners should use condoms during intercourse for the duration of the study and until the end of estimated relevant potential exposure in WOCBP (90 days).
- Men must agree to refrain from sperm donation for the duration of the study and until their plasma concentrations are below the level that could result in a relevant potential exposure to a possible fetus, predicted to be 90 days following last dose of study drug.
- Men who are in exclusively same-sex relationships (as their preferred and usual lifestyle) are not required to use contraception.

4b. Female participants:

- Women of childbearing potential who are abstinent (if this was complete abstinence, as their preferred and usual lifestyle) or in a same-sex relationship (as part of their preferred and usual lifestyle) must agree to either remain abstinent or stay in a same-sex relationship without sexual relationships with males. Periodic abstinence (e.g., calendar, ovulation, symptothermal, postovulation methods), declaration of abstinence just for the duration of a study, and withdrawal are not acceptable methods of contraception.
- Otherwise, WOCBP participating must agree to use effective contraception, (see contraception guidance below), for the entirety of the study. Contraception must continue following completion of study drug administration for 30 days.
  - Women of childbearing potential participating must test negative for pregnancy prior to initiation of treatment as indicated by a negative serum pregnancy test at the screening visit followed by a negative urine pregnancy test within 48 hours prior to exposure and at other times.
  - Two forms of effective contraception, where at least 1 form was highly effective (less than 1% failure rate, such as combination oral contraceptives, implanted contraceptives, or intrauterine devices) will be used. Effective contraception (such as male or female condoms with spermicide, diaphragms with spermicide, or cervical sponges) may be used as the second therapy. Barrier protection methods without concomitant use of a spermicide are not a reliable or acceptable method. Thus, each barrier method must include use of a spermicide (that is, condom with spermicide, diaphragm with spermicide, female condom with spermicide). It should be noted that the use of male and female condoms as a double-barrier method was not considered acceptable due to the high failure rate when these methods are combined.
- Women who are not of childbearing potential may participate and include those who are:
  - Infertile due to surgical sterilization (hysterectomy, bilateral oophorectomy, or tubal ligation), congenital anomaly such as mullerian agenesis, or
  - Postmenopausal – defined as either
    1. A woman at least 50 years of age with an intact uterus, not on hormone therapy, who has had either
       1. cessation of menses for at least 1 year, or
       2. at least 6 months of spontaneous amenorrhea with a follicle-stimulating hormone >40 mIU/mL; or
    2. A woman at least 55 years of age not on hormone therapy, who has had at least 6 months of spontaneous amenorrhea; or
    3. A woman at least 55 years of age with a diagnosis of menopause prior to starting hormone replacement therapy.

1. Have a body mass index between 23.0 kg/m^2^ and 45.0 kg/m^2^, inclusive, at screening; are of stable weight (±5%) >3 months prior to screening; and agree not to initiate an intensive diet and/or exercise program during the study with the intent of reducing body weight other than lifestyle and dietary measures for diabetes treatment;
2. Have venous access sufficient to allow for blood sampling as per the protocol.

**Informed Consent**

1. Are reliable and willing to make themselves available for the duration of the study and are willing to follow study procedures;
2. Have given written informed consent approved by Lilly and the ethical review board (ERB) governing the site.

**Exclusion Criteria**

Participants will be excluded from study enrollment if they meet any of the following criteria at screening and/or at other visits prior to randomization, when indicated:

**Primary Study Condition - Diabetes Related**

1. Have type 1 diabetes mellitus;
2. Have had more than 1 episode of severe hypoglycemia, as defined by the American Diabetes Association criteria, within 6 months before screening or has a history of hypoglycemia unawareness or poor recognition of hypoglycemic symptoms; any participants that cannot communicate an understanding of hypoglycemic symptoms and the appropriate treatment of hypoglycemia prior to the first dose of study drug should also be excluded;
3. Have had 1 or more episodes of ketoacidosis or hyperosmolar state/coma requiring hospitalization within the 6 months prior to screening;
4. Have a history of proliferative retinopathy or maculopathy as determined by the investigator based on a recent (<6 months) ophthalmologic examination;
5. Impaired renal estimated glomerular filtration rate <60 mL/min/1.73 m^2^ calculated by Chronic Kidney Disease-Epidemiology. One retest may be performed in case of an initial result <60 mL/min/1.73 m^2^. The highest value from the 2 tests will be accepted.

**Prior/Concomitant Therapy - Glucose-Lowering Medications**

1. Have taken any glucose-lowering medications other than those indicated in Inclusion Criterion [2] during the last 3 months before screening or during the screening/lead-in period; short-term use of insulin (<14 days) for treatment of acute conditions was allowed in the 3-month period prior to entry and after randomization

**Medical Conditions - General**

1. Have a history or current cardiovascular, respiratory, hepatic, renal, GI, endocrine, hematological, or neurological disorders capable of significantly altering the absorption, metabolism, or elimination of drugs; of constituting a risk when taking the IP; or of interfering with the interpretation of data
2. Have acute or chronic pancreatitis or a history of acute idiopathic pancreatitis; participants who had cholecystolithiasis and/or cholecystectomy in the past, with no long-term complications, are eligible for participation;
3. Have a known clinically significant gastric emptying abnormality (e.g., severe diabetic gastroparesis or gastric outlet obstruction) or have undergone gastric bypass (bariatric) surgery or restrictive bariatric surgery (e.g., Lap-Band®);
4. Have a personal or family history of medullary thyroid carcinoma (MTC), multiple endocrine neoplasia syndrome type 2 (MEN 2), or calcitonin ≥20 pg/mL (≥5.85 pmol/L) at screening;
5. Have had acute myocardial infarction, congestive heart failure New York Heart Association Class III or IV, history of or suspected ischemic heart disease, and/or cerebrovascular accident (stroke [including transient ischemic attack]);
6. Have findings in the 12-lead ECG at screening that, in the opinion of the investigator, may increase the risks of potentially clinically relevant worsening associated with participation in the study;
7. Have an active or untreated malignancy or have been in remission from a clinically significant malignancy (other than basal or squamous cell skin cancer, in situ carcinomas of the cervix, or in situ prostate cancer) for <5 years prior to screening;
8. Have evidence of human immunodeficiency virus (HIV) and/or positive HIV antibodies;
9. Have evidence of hepatitis B or positive hepatitis B surface antigen and/or evidence of hepatitis C virus (HCV) or hepatitis C antibody at screening. Participants with a previous diagnosis of HCV who have been treated with antiviral therapy and achieved a sustained virological response may be eligible for inclusion in the study, provided they have no detectable HCV RNA on the screening HCV polymerase chain reaction test. A sustained virological response was defined as an undetectable HCV RNA level 24 weeks after completion of a full, documented course of an approved antiviral therapy for HCV. Participants who have spontaneously cleared HCV infection, defined as (1): a positive HCV antibody test and (2): a negative HCV RNA test, with no history of anti-HCV treatment, may be eligible for inclusion in the study, provided they have no detectable HCV RNA on screening for this study.
10. Have serum aspartate aminotransferase (AST) or alanine aminotransferase (ALT) >2.5× the upper limit of normal (ULN) or total bilirubin level (TBL) >1.5× ULN;
11. Have had a blood donation of 500 mL or more in the last 3 months or any blood donation within the last month prior to screening;
12. Have had a blood transfusion or severe blood loss within the last 3 months or have known hemoglobinopathy, hemolytic anemia, sickle cell anemia, or have a hemoglobin value <11 g/dL (males) or <10 g/dL (females), or any other condition known to interfere with hemoglobin A1c measurement;
13. Have a history of drug or alcohol abuse; and/or smoke >10 cigarettes per day or the equivalent; or are unable or unwilling to refrain from nicotine 4 hours before each CRU admission and outpatient visit, and throughout the duration of each CRU visit;
14. Have an average weekly alcohol intake that exceeds 21 units per week (males) and 14 units per week (females) (1 unit = 12 oz or 360 mL of beer; 5 oz or 150 mL of wine; 1.5 oz or 45 mL of distilled spirits), or are unwilling to stop alcohol consumption 24 hours before each CRU admission and outpatient visit, and throughout the duration of each CRU visit;
15. Have evidence of significant active neuropsychiatric disease as determined by the investigator;

**Prior/Concomitant Therapy - General**

1. Have been treated with prescription drugs that promote weight loss (e.g., sibutramine, mazindol, phentermine, lorcaserin, naltrexone/bupropion, liraglutide) or similar other body weight loss medications, including over-the-counter medications (e.g., alli®) within 3 months prior to screening or between screening and randomization;
2. Have received chronic (lasting >14 consecutive days) systemic glucocorticoid therapy (excluding topical, intra-articular, and inhaled preparations) within 1 month before screening, or between screening and randomization;
3. Have received treatment with a drug that has not received regulatory approval for any indication within 1 month prior to screening; if the previous study drug has a long half-life, 3 months or 5 half-lives (whichever was longer) should have passed.

**Prior/Concurrent Clinical Trial Experience**

1. Are persons who have previously completed or withdrawn from this study;
2. Have previous exposure or known allergies to tirzepatide or related compounds, or have an intolerance to GLP-1 RAs;
3. Are currently enrolled in a clinical study involving an IP or any other type of medical research judged not to be scientifically or medically compatible with this study.

**Other Exclusions**

1. Are investigative site personnel directly affiliated with this study and their immediate families. Immediate family was defined as a spouse, parent, child, or sibling, whether biological or legally adopted;
2. Are Eli Lilly and Company, University of Graz, or Covance employees;
3. Are deemed unsuitable by the investigator for any other reason.

**Definitions of Hypoglycemia**

***Glucose Alert Value (Level 1):***

- **Documented symptomatic hypoglycemia** was defined as any time a participants feels that he or she was experiencing symptoms and/or signs associated with hypoglycemia and has a PG level of ≤3.9 mmol/L (≤70 mg/dL).
- **Documented asymptomatic hypoglycemia** was defined as any event not accompanied by typical symptoms of hypoglycemia, but with a measured PG ≤3.9 mmol/L (≤70 mg/dL).
- **Documented unspecified hypoglycemia** was defined as any event with no

information about symptoms of hypoglycemia available, but with a measured PG ≤3.9 mmol/L (≤70 mg/dL).

***Clinically Significant Hypoglycemia (Level 2):***

- **Documented symptomatic hypoglycemia** was defined as any time a participants feels that he/she was experiencing symptoms and/or signs associated with hypoglycemia and has a PG level of <3.0 mmol/L (<54 mg/dL).
- **Documented asymptomatic hypoglycemia** was defined as any event not accompanied by typical symptoms of hypoglycemia, but with a measured PG <3.0 mmol/L (<54 mg/dL).
- **Documented unspecified hypoglycemia** was defined as any event with no information about symptoms of hypoglycemia available, but with a measured PG <3.0 mmol/L (<54 mg/dL).

***Severe Hypoglycemia (Level 3):***

**Severe hypoglycemia** was defined as an episode with severe cognitive impairment requiring the assistance of another person to actively administer carbohydrate, glucagon, or other resuscitative actions. These episodes may be associated with sufficient neuroglycopenia to induce seizure or coma. Plasma glucose measurements may not be available during such an event, but neurological recovery attributable to the restoration of BG to normal was considered sufficient evidence that the event was induced by a low BG concentration.

***Other Hypoglycemia Categories:***

**Nocturnal hypoglycemia** was defined as any hypoglycemic event that occurs between bedtime and waking.

**SUPPLEMENTAL TABLES**

**Table S1. Dose Escalation Schemes**

|  | **Dose Escalation Schemes** | | | |
| --- | --- | --- | --- | --- |
| **Treatment** | Weeks 1-2 | Weeks 3-4 | Weeks 5-8 | Weeks 9-12 |
| Tirzepatide | 1 × 2.5-mg PFS | 1 × 5-mg PFS | 1 × 10-mg PFS | 1 × 15-mg PFS |
| Placebo | 1 × PFS | 1 × PFS | 1 × PFS | 1 × PFS |

A once weekly dose of 2.5 mg was initiated for two weeks, followed by an increase to once weekly tirzepatide 5 mg for two weeks, and once weekly tirzepatide 10 mg for 4 weeks until the 15-mg dose was reached and maintained for the remainder of the treatment period (4 weeks). Abbreviations: PFS=prefilled syringe.

**Table S2. Key time points for hypoglycemic clamp**

| **Clamp plasma glucose level** | **Planned key time points** | **Clamp stage comment** |
| --- | --- | --- |
| 100 mg/dL (5.5 mmol/L) | -30 minutes | Start of 100 mg/dL (5.5 mmol/L) plateau |
| End of 100 mg/dL (5.5 mmol/L) | 0 minutes | End of 100 mg/dL (5.5 mmol/L) plateau |
| Reaching 63 mg/dL (3.5 mmol/L) | *I* minutes | Start of 63 mg/dL (3.5 mmol/L) plateau |
| End of 63 mg/dL (3.5 mmol/L) | *I* + 30 minutes | End of 63 mg/dL (3.5 mmol/L) plateau |
| Reaching 45 mg/dL (nadir, 2.5 mmol/L) | *J* minutes | Start of nadir plateau  End of constant insulin infusion  Start recovery |
| End of 45 mg/dL (nadir, 2.5 mmol/L) | *J* + 30 minutes | End of nadir plateau |
| Reaching 72 mg/dL (4.0 mmol/L) | *K* minutes | Time point 72 mg/dL (4.0 mmol/L) reached |

Note: The plasma glucose level and duration of each plateau could have varied due to the participant’s condition.

**Table S3. Hypoglycemic symptom scores during induced hypoglycemia and recovery**

| **Parameter** | **Target PG** | **Treatment arm** | **n** | **LSM (SE)** | **Difference in LSM (95% CI) [p-value]** |
| --- | --- | --- | --- | --- | --- |
| **Neuroglycopenic symptoms (cognitive dysfunction)** | **100 mg/dL (5.5 mmol/L)** | Placebo | 33 | 1.06 (0.02) |  |
|  |  | Tirzepatide 15 mg | 33 | 1.03 (0.02) | -0.04 (-0.08, 0.00) [0.0577] |
|  | **63 mg/dL (3.5 mmol/L)** | Placebo | 33 | 1.16 (0.04) |  |
|  |  | Tirzepatide 15 mg | 33 | 1.06 (0.04) | -0.11 (-0.21, 0.00) [0.0463] |
|  | **45 mg/dL (2.5 mmol/L)** | Placebo | 31 | 1.20 (0.05) |  |
|  |  | Tirzepatide 15 mg | 33 | 1.17 (0.05) | -0.03 (-0.13, 0.07) [0.5540] |
|  | **72 mg/dL (4.0 mmol/L)** | Placebo | 33 | 1.13 (0.05) |  |
|  |  | Tirzepatide 15 mg | 33 | 1.10 (0.05) | -0.04 (-0.12, 0.05) [0.3798] |
| **Neuroglycopenic symptoms (neuroglycopenia)** | **100 mg/dL (5.5 mmol/L)** | Placebo | 33 | 1.45 (0.09) |  |
|  |  | Tirzepatide 15 mg | 33 | 1.31 (0.09) | -0.14 (-0.33, 0.05) [0.1379] |
|  | **63 mg/dL (3.5 mmol/L)** | Placebo | 33 | 1.69 (0.11) |  |
|  |  | Tirzepatide 15 mg | 33 | 1.38 (0.11) | -0.31 (-0.56, -0.06) [0.0171] |
|  | **45 mg/dL (2.5 mmol/L)** | Placebo | 31 | 2.09 (0.15) |  |
|  |  | Tirzepatide 15 mg | 33 | 1.81 (0.15) | -0.28 (-0.52, -0.04) [0.0222] |
|  | **72 mg/dL (4.0 mmol/L)** | Placebo | 33 | 2.06 (0.16) |  |
|  |  | Tirzepatide 15 mg | 33 | 1.78 (0.16) | -0.28 (-0.59, 0.04) [0.0845] |
| **Autonomic symptoms** | **100 mg/dL (5.5 mmol/L)** | Placebo | 33 | 1.09 (0.03) | -0.01 (-0.09, 0.07) [0.7998] |
|  |  | Tirzepatide 15 mg | 33 | 1.08 (0.03) |  |
|  | **63 mg/dL (3.5 mmol/L)** | Placebo | 33 | 1.26 (0.06) | -0.15 (-0.30, 0.00) [0.0575] |
|  |  | Tirzepatide 15 mg | 33 | 1.11 (0.06) |  |
|  | **45 mg/dL (2.5 mmol/L)** | Placebo | 31 | 2.09 (0.13) | -0.36 (-0.60, -0.12) [0.0054] |
|  |  | Tirzepatide 15 mg | 33 | 1.73 (0.13) |  |
|  | **72 mg/dL (4.0 mmol/L)** | Placebo | 33 | 1.48 (0.14) | -0.05 (-0.38, 0.29) [0.7718] |
|  |  | Tirzepatide 15 mg | 33 | 1.44 (0.14) |  |
| **Overall score** | **100 mg/dL (5.5 mmol/L)** | Placebo | 33 | 1.19 (0.03) | -0.06 (-0.13, 0.00) [0.0505] |
|  |  | Tirzepatide 15 mg | 33 | 1.13 (0.03) |  |
|  | **63 mg/dL (3.5 mmol/L)** | Placebo | 33 | 1.35 (0.06) | -0.18 (-0.31, -0.05) [0.0104] |
|  |  | Tirzepatide 15 mg | 33 | 1.17 (0.06) |  |
|  | **45 mg/dL (2.5 mmol/L)** | Placebo | 31 | 1.68 (0.08) | -0.19 (-0.32, -0.06) [0.0068] |
|  |  | Tirzepatide 15 mg | 33 | 1.49 (0.08) |  |
|  | **72 mg/dL (4.0 mmol/L)** | Placebo | 33 | 1.50 (0.09) | -0.11 (-0.30, 0.08) [0.2302] |
|  |  | Tirzepatide 15 mg | 33 | 1.38 (0.09) |  |

Abbreviations: CI=confidence interval; LSM=least squares mean; PG=plasma glucose; SE=standard error.

**Table S4. Individual symptom scores during induced hypoglycemia and recovery**

| **Parameter** | **Target PG** | **Treatment arm** | **n** | **LSM (SE)** | **Difference in LSM (95% CI) [p-value]** |
| --- | --- | --- | --- | --- | --- |
| **Inability to concentrate** | **100 mg/dL (5.5 mmol/L)** | Placebo | 33 | 1.14 (0.06) |  |
|  |  | Tirzepatide 15 mg | 33 | 1.04 (0.06) | -0.10 (-0.22, 0.02) [0.0863] |
|  | **63 mg/dL (3.5 mmol/L)** | Placebo | 33 | 1.29 (0.08) |  |
|  |  | Tirzepatide 15 mg | 33 | 1.04 (0.08) | -0.25 (-0.48, -0.02) [0.0337] |
|  | **45 mg/dL (2.5 mmol/L)** | Placebo | 31 | 1.29 (0.09) |  |
|  |  | Tirzepatide 15 mg | 33 | 1.23 (0.09) | -0.06 (-0.21, 0.09) [0.4408] |
|  | **72 mg/dL (4.0 mmol/L)** | Placebo | 33 | 1.14 (0.09) |  |
|  |  | Tirzepatide 15 mg | 33 | 1.23 (0.09) | 0.09 (-0.14, 0.32) [0.4332] |
| **Blurred vision** | **100 mg/dL (5.5 mmol/L)** | Placebo | 33 | 1.08 (0.04) |  |
|  |  | Tirzepatide 15 mg | 33 | 1.06 (0.04) | -0.02 (-0.11, 0.07) [0.6770] |
|  | **63 mg/dL (3.5 mmol/L)** | Placebo | 33 | 1.24 (0.08) |  |
|  |  | Tirzepatide 15 mg | 33 | 1.17 (0.08) | -0.07 (-0.22, 0.07) [0.3121] |
|  | **45 mg/dL (2.5 mmol/L)** | Placebo | 31 | 1.22 (0.11) |  |
|  |  | Tirzepatide 15 mg | 33 | 1.33 (0.11) | 0.10 (-0.10, -0.30) [0.3045] |
|  | **72 mg/dL (4.0 mmol/L)** | Placebo | 33 | 1.16 (0.07) |  |
|  |  | Tirzepatide 15 mg | 33 | 1.12 (0.07) | -0.03 (-0.18, 0.11) [0.6360] |
| **Anxiety** | **100 mg/dL (5.5 mmol/L)** | Placebo | 33 | 1.00 (0.02) | 0.03 (-0.03, 0.09) [0.2760] |
|  |  | Tirzepatide 15 mg | 33 | 1.03 (0.02) |  |
|  | **63 mg/dL (3.5 mmol/L)** | Placebo | 33 | 1.03 (0.02) | -0.03 (-0.09, 0.03) [0.3661] |
|  |  | Tirzepatide 15 mg | 33 | 1.00 (0.02) |  |
|  | **45 mg/dL (2.5 mmol/L)** | Placebo | 31 | 1.02 (0.04) | 0.06 (-0.60, 0.18) [0.3245] |
|  |  | Tirzepatide 15 mg | 33 | 1.08 (0.04) |  |
|  | **72 mg/dL (4.0 mmol/L)** | Placebo | 33 | NC | NC |
|  |  | Tirzepatide 15 mg | 33 | NC |  |
| **Confusion** | **100 mg/dL (5.5 mmol/L)** | Placebo | 33 | 1.01 (0.01) | -0.01 (-0.04, 0.02) [0.3661] |
|  |  | Tirzepatide 15 mg | 33 | 1.00 (0.01) |  |
|  | **63 mg/dL (3.5 mmol/L)** | Placebo | 33 | 1.10 (0.05) | -0.10 (-0.23, 0.03) [0.1267] |
|  |  | Tirzepatide 15 mg | 33 | 1.00 (0.05) |  |
|  | **45 mg/dL (2.5 mmol/L)** | Placebo | 31 | 1.18 (0.06) | -0.13 (-0.30, 0.04) [0.1272] |
|  |  | Tirzepatide 15 mg | 33 | 1.06 (0.06) |  |
|  | **72 mg/dL (4.0 mmol/L)** | Placebo | 33 | 1.22 (0.08) | -0.19 (-0.41, 0.03) [0.0922] |
|  |  | Tirzepatide 15 mg | 33 | 1.03 (0.08) |  |
| **Difficulty speaking** | **100 mg/dL (5.5 mmol/L)** | Placebo | 33 | 1.09 (0.05) |  |
|  |  | Tirzepatide 15 mg | 33 | 1.01 (0.05) | -0.08 (-0.21, 0.05) [0.2266] |
|  | **63 mg/dL (3.5 mmol/L)** | Placebo | 33 | 1.16 (0.05) |  |
|  |  | Tirzepatide 15 mg | 33 | 1.04 (0.05) | -0.12 (-0.26, 0.01) [0.0764] |
|  | **45 mg/dL (2.5 mmol/L)** | Placebo | 31 | 1.24 (0.08) |  |
|  |  | Tirzepatide 15 mg | 33 | 1.16 (0.08) | -0.09 (-0.30, 0.12) [0.3865] |
|  | **72 mg/dL (4.0 mmol/L)** | Placebo | 33 | 1.16 (0.09) |  |
|  |  | Tirzepatide 15 mg | 33 | 1.12 (0.09) | -0.03 (-0.18, 0.11) [0.6360] |
| **Double vision** | **100 mg/dL (5.5 mmol/L)** | Placebo | 33 | 1.06 (0.03) |  |
|  |  | Tirzepatide 15 mg | 33 | 1.01 (0.03) | -0.05 (-0.13, 0.03) [0.2409] |
|  | **63 mg/dL (3.5 mmol/L)** | Placebo | 33 | 1.15 (0.06) |  |
|  |  | Tirzepatide 15 mg | 33 | 1.09 (0.06) | -0.06 (-0.18, 0.05) [0.2738] |
|  | **45 mg/dL (2.5 mmol/L)** | Placebo | 31 | 1.16 (0.07) |  |
|  |  | Tirzepatide 15 mg | 33 | 1.15 (0.07) | -0.01 (-0.16, 0.14) [0.9380] |
|  | **72 mg/dL (4.0 mmol/L)** | Placebo | 33 | 1.12 (0.06) |  |
|  |  | Tirzepatide 15 mg | 33 | 1.07 (0.06) | -0.06 (-0.18, 0.07) [0.3696] |
| **Drowsiness** | **100 mg/dL (5.5 mmol/L)** | Placebo | 33 | 1.03 (0.03) |  |
|  |  | Tirzepatide 15 mg | 33 | 1.04 (0.03) | 0.01 (-0.06, 0.09) [0.7252] |
|  | **63 mg/dL (3.5 mmol/L)** | Placebo | 33 | 1.38 (0.10) |  |
|  |  | Tirzepatide 15 mg | 33 | 1.11 (0.10) | -0.27 (-0.54, 0.00) [0.0498] |
|  | **45 mg/dL (2.5 mmol/L)** | Placebo | 31 | 1.62 (0.12) |  |
|  |  | Tirzepatide 15 mg | 33 | 1.38 (0.12) | -0.24 (-0.52, 0.03) [0.0809] |
|  | **72 mg/dL (4.0 mmol/L)** | Placebo | 33 | 1.37 (0.11) |  |
|  |  | Tirzepatide 15 mg | 33 | 1.26 (0.11) | -0.11 (-0.36, 0.14) [0.3696] |
| **Tiredness** | **100 mg/dL (5.5 mmol/L)** | Placebo | 33 | 1.86 (0.18) |  |
|  |  | Tirzepatide 15 mg | 33 | 1.59 (0.18) | -0.26 (-0.52, 0.00) [0.0503] |
|  | **63 mg/dL (3.5 mmol/L)** | Placebo | 33 | 2.08 (0.19) |  |
|  |  | Tirzepatide 15 mg | 33 | 1.77 (0.19) | -0.31 (-0.66, 0.04) [0.0845] |
|  | **45 mg/dL (2.5 mmol/L)** | Placebo | 31 | 2.39 (0.24) |  |
|  |  | Tirzepatide 15 mg | 33 | 2.19 (0.23) | -0.20 (-0.73, 0.34) [0.4566] |
|  | **72 mg/dL (4.0 mmol/L)** | Placebo | 33 | 2.33 (0.26) |  |
|  |  | Tirzepatide 15 mg | 33 | 2.09 (0.26) | -0.23 (-0.83, 0.37) [0.4335] |
| **Hunger** | **100 mg/dL (5.5 mmol/L)** | Placebo | 33 | 1.72 (0.22) |  |
|  |  | Tirzepatide 15 mg | 33 | 1.45 (0.22) | -0.27 (-0.83, 0.28) [0.3251] |
|  | **63 mg/dL (3.5 mmol/L)** | Placebo | 33 | 1.91 (0.21) |  |
|  |  | Tirzepatide 15 mg | 33 | 1.48 (0.21) | -0.44 (-0.90, 0.03) [0.0641] |
|  | **45 mg/dL (2.5 mmol/L)** | Placebo | 31 | 2.62 (0.24) |  |
|  |  | Tirzepatide 15 mg | 33 | 1.99 (0.24) | -0.62 (-1.00, -0.24) [0.0022] |
|  | **72 mg/dL (4.0 mmol/L)** | Placebo | 33 | 3.14 (0.30) |  |
|  |  | Tirzepatide 15 mg | 33 | 2.34 (0.30) | -0.81 (-1.34, -0.27) [0.0047] |
| **Weakness** | **100 mg/dL (5.5 mmol/L)** | Placebo | 33 | 1.19 (0.08) |  |
|  |  | Tirzepatide 15 mg | 33 | 1.15 (0.08) | -0.04 (-0.16, 0.08) [0.5231] |
|  | **63 mg/dL (3.5 mmol/L)** | Placebo | 33 | 1.38 (0.09) |  |
|  |  | Tirzepatide 15 mg | 33 | 1.15 (0.09) | -0.22 (-0.49, 0.04) [0.0970] |
|  | **45 mg/dL (2.5 mmol/L)** | Placebo | 31 | 1.71 (0.15) |  |
|  |  | Tirzepatide 15 mg | 33 | 1.67 (0.15) | -0.04 (-0.26, 0.19) [0.7273] |
|  | **72 mg/dL (4.0 mmol/L)** | Placebo | 33 | 1.38 (0.15) |  |
|  |  | Tirzepatide 15 mg | 33 | 1.43 (0.15) | 0.05 (-0.30, 0.40) [0.7711] |
| **Sweating** | **100 mg/dL (5.5 mmol/L)** | Placebo | 33 | 1.03 (0.03) |  |
|  |  | Tirzepatide 15 mg | 33 | 1.03 (0.03) | 0.00 (-0.09, 0.09) [1.0000] |
|  | **63 mg/dL (3.5 mmol/L)** | Placebo | 33 | 1.16 (0.05) |  |
|  |  | Tirzepatide 15 mg | 33 | 1.04 (0.05) | -0.12 (-0.27, 0.02) [0.0998] |
|  | **45 mg/dL (2.5 mmol/L)** | Placebo | 31 | 2.43 (0.19) |  |
|  |  | Tirzepatide 15 mg | 33 | 1.94 (0.19) | -0.49 (-0.82, -0.16) [0.0048] |
|  | **72 mg/dL (4.0 mmol/L)** | Placebo | 33 | 1.62 (0.19) |  |
|  |  | Tirzepatide 15 mg | 33 | 1.47 (0.19) | -0.14 (-0.63, 0.34) [0.5480] |
| **Trembling** | **100 mg/dL (5.5 mmol/L)** | Placebo | 33 | 1.00 (0.02) |  |
|  |  | Tirzepatide 15 mg | 33 | 1.04 (0.02) | 0.04 (-0.02, 0.11) [0.2155] |
|  | **63 mg/dL (3.5 mmol/L)** | Placebo | 33 | 1.09 (0.05) |  |
|  |  | Tirzepatide 15 mg | 33 | 1.11 (0.05) | 0.03 (-0.12, 0.17) [0.7203] |
|  | **45 mg/dL (2.5 mmol/L)** | Placebo | 31 | 1.41 (0.12) |  |
|  |  | Tirzepatide 15 mg | 33 | 1.39 (0.12) | -0.03 (-0.22, 0.16) [0.7624] |
|  | **72 mg/dL (4.0 mmol/L)** | Placebo | 33 | 1.18 (0.11) |  |
|  |  | Tirzepatide 15 mg | 33 | 1.25 (0.11) | 0.07 (-0.24, 0.39) [0.6493] |
| **Warmness** | **100 mg/dL (5.5 mmol/L)** | Placebo | 33 | 1.24 (0.09) |  |
|  |  | Tirzepatide 15 mg | 33 | 1.17 (0.09) | -0.07 (-0.24, 0.10) [0.3916] |
|  | **63 mg/dL (3.5 mmol/L)** | Placebo | 33 | 1.53 (0.12) |  |
|  |  | Tirzepatide 15 mg | 33 | 1.18 (0.12) | -0.34 (-0.64, -0.05) [0.0219] |
|  | **45 mg/dL (2.5 mmol/L)** | Placebo | 31 | 2.42 (0.19) |  |
|  |  | Tirzepatide 15 mg | 33 | 1.85 (0.19) | -0.57 (-0.98, -0.16) [0.0079] |
|  | **72 mg/dL (4.0 mmol/L)** | Placebo | 33 | 1.66 (0.21) |  |
|  |  | Tirzepatide 15 mg | 33 | 1.58 (0.21) | -0.07 (-0.39, 0.25) [0.6509] |

Abbreviations: CI=confidence interval; LSM=least squares mean; NC=not calculable; PG=plasma glucose; SE=standard error.

**Table S5. Safety profile**

| **Parameter** | **Number of events [number of participants with events]**  **(percent of participants reporting events)** | |
| --- | --- | --- |
|  | **Placebo  N=36** | **Tirzepatide 15 mg  N=39** |
| **All TEAEs** | 40 [18] (50.0%) | 132 [30] (76.9%) |
| **Treatment-related TEAEs** | 9 [6] (16.7%) | 94 [24] (61.5%) |
| **Deaths** | 0 [0] (0.0%) | 0 [0] (0.0%) |
| **SAEs^a^** | 0 [0] (0.0%) | 4 [4] (10.3%) |
| **Treatment-related SAEs** | 0 [0] (0.0%) | 0 [0] (0.0%) |
| **AEs leading to discontinuation from study^b^** | 1 [1] (2.8%) | 2 [2] (5.1%) |
| **AEs of special interest^c^** | 0 [0] (0.0%) | 6 [2] (5.1%) |

Safety population.

^a^ SAEs included facial paralysis, obstructive pancreatitis, scrotal abscess, and hospitalization due to a preexisting condition of intervertebral disc protrusion.

^b^ One participant completed Period 1, in which they received tirzepatide, but was discontinued during washout due to an SAE of obstructive pancreatitis, one participant discontinued in Period 1 after receiving seven doses of tirzepatide due to nausea and one participant was discontinued in Period 1 after receiving 10 doses of placebo due to coronary artery disease.

^c^ AEs of special interest included severe obstructive pancreatitis and atrial fibrillation reported by one participant, which were unrelated to study drug, and severe upper abdominal pain, vomiting, nausea, and syncope reported by one participant, which were considered related to study drug.

Abbreviations: AEs=adverse events; SAEs=serious adverse events; TEAEs=treatment-emergent adverse events.

**SUPPLEMENTAL FIGURES**

**Figure S1. Participant disposition**


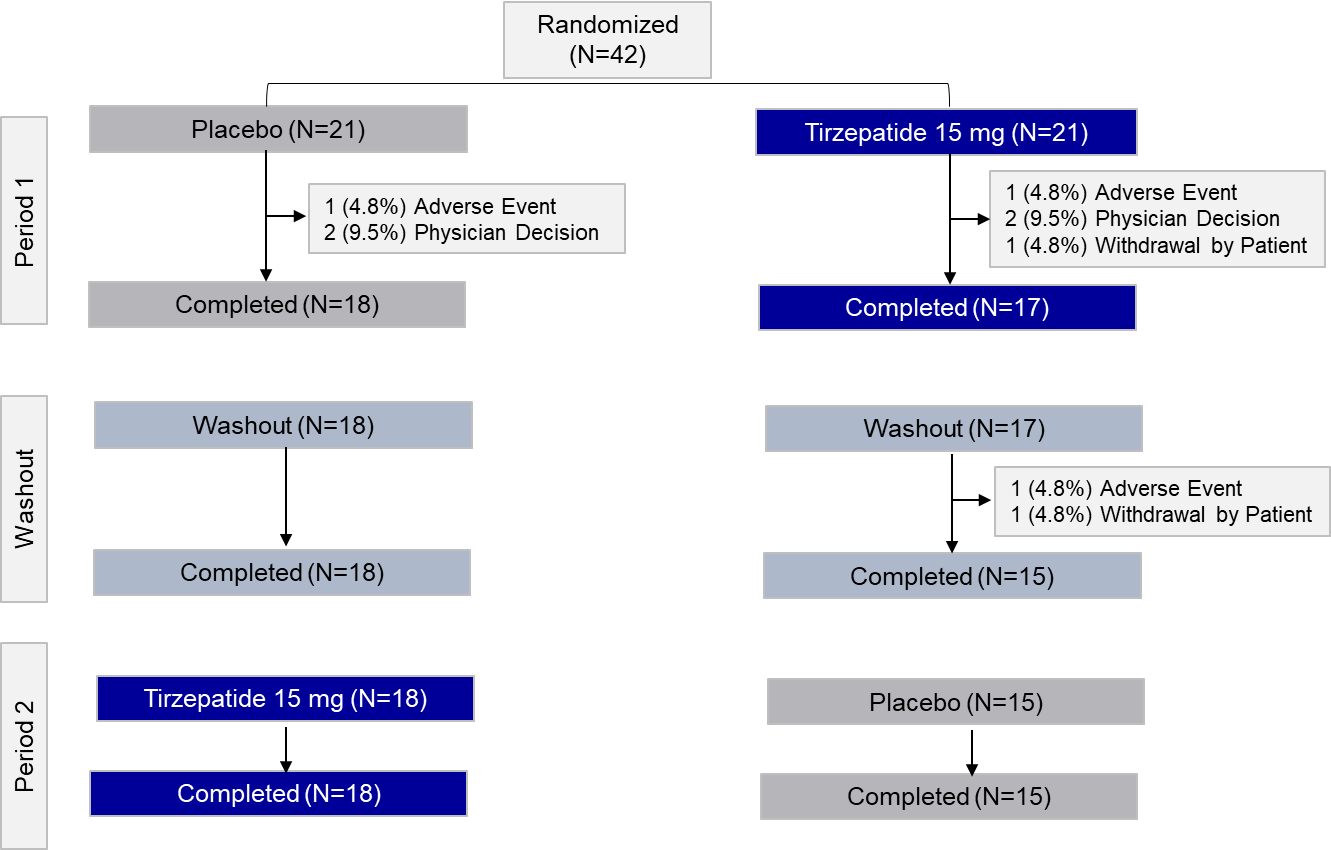


Percentages are based on the number of participants randomized.

**Figure S2. Glucose infusion-related parameters during induced hypoglycemia**


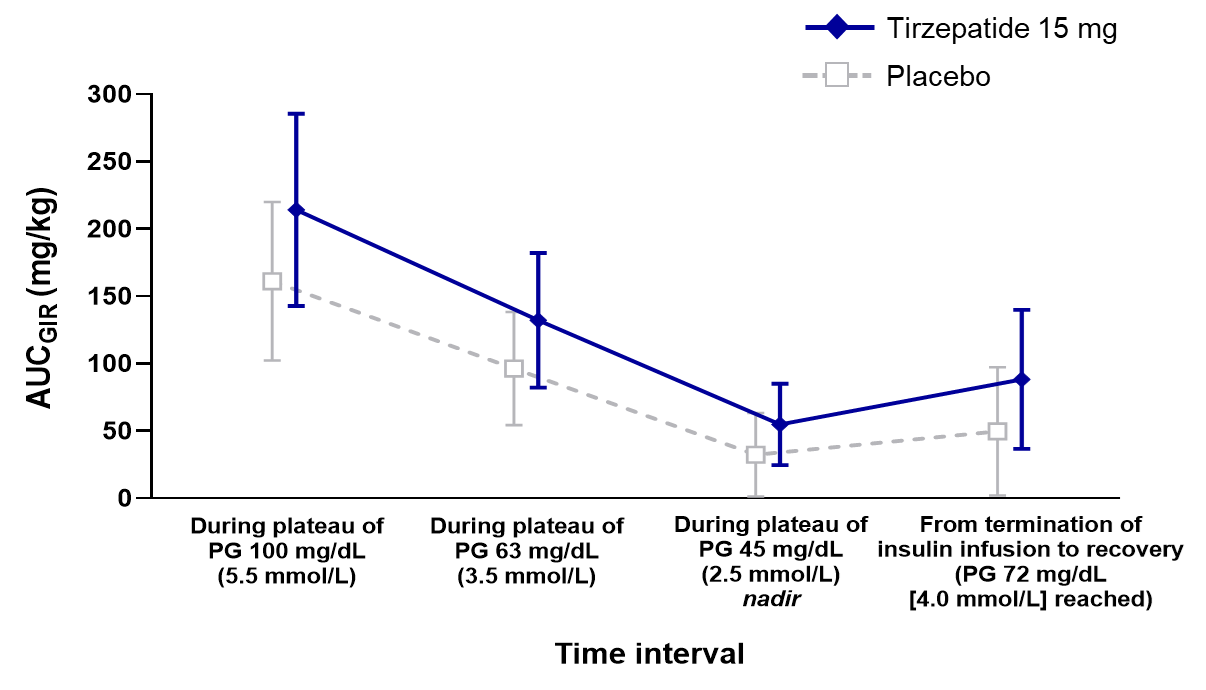


Data are mean (SD). Pharmacodynamic population. LSM treatment differences (95% confidence intervals) in AUC_GIR_ were 51.05 mg/kg (23.19, 78.92) [p=0.0008] during plateau of PG 100 mg/dL (5.5 mmol/L), 36.25 mg/kg (17.21, 55.28) [p=0.0005] during plateau of PG 63 mg/dL (3.5 mmol/L), and 22.37 mg/kg (11.94, 32.79) [p=0.0001] during plateau of PG 45 mg/dL (nadir, 2.5 mmol/L). Abbreviations: AUC_GIR_=area under the curve of glucose infusion rate; LSM=least squares mean; PG=plasma glucose; SD=standard deviation.

**Figure S3. Hypoglycemia awareness during clamp-induced hypoglycemia**

Data are percent of participants aware of hypoglycemia. Pharmacodynamic population. Hypoglycemia awareness was evaluated based on participant response (yes/no) to question "Do you feel hypoglycemic" during induced hypoglycemia. If the participant answered "yes" at least once when the question was asked twice in the same plateau, the participant was aware of hypoglycemia. Abbreviations: PG=plasma glucose.

**Figure S4. Vital signs during induced hypoglycemia**

**
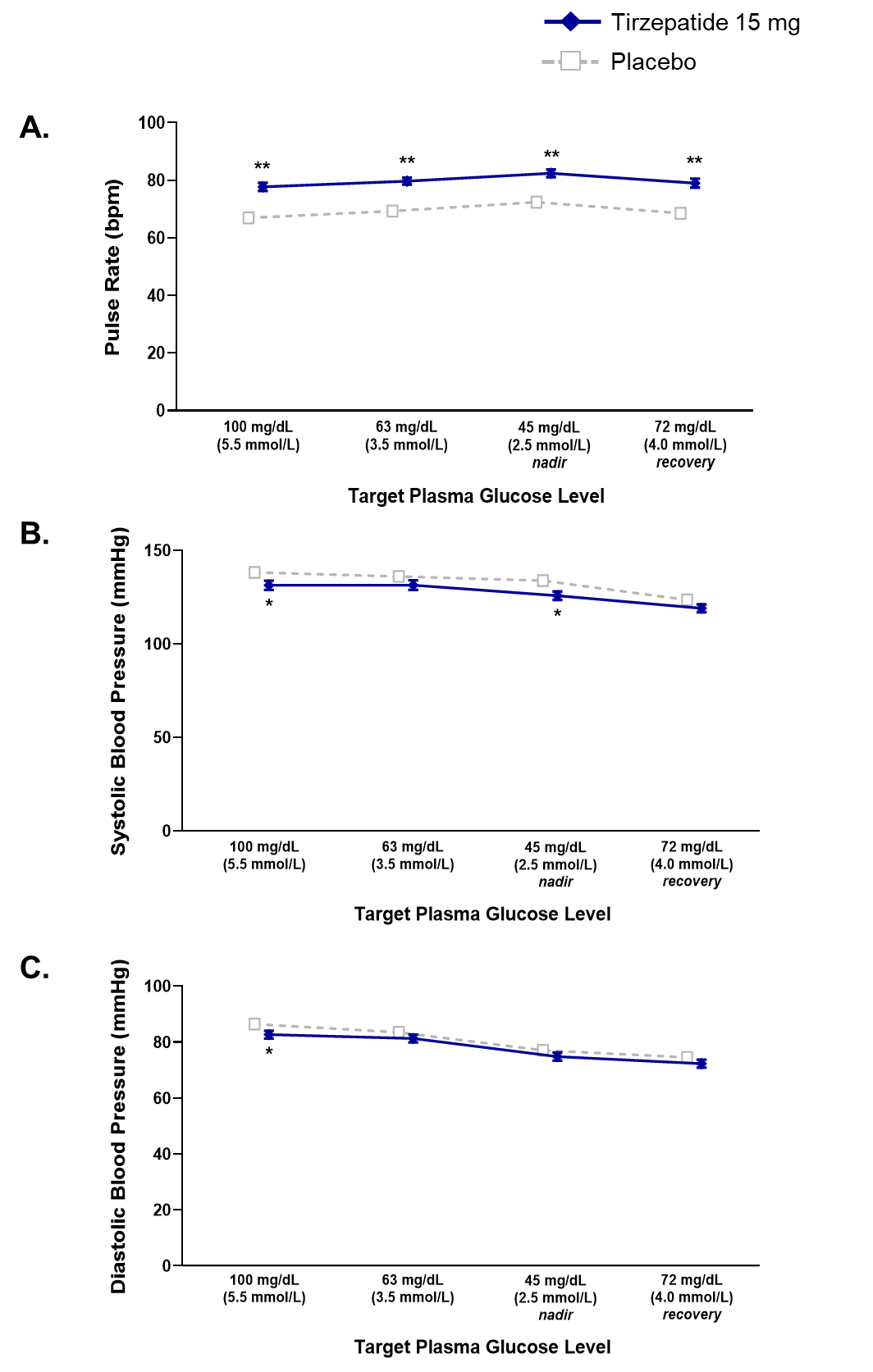
**

Data are LSM (SE). Pharmacodynamic population. *p<0.05 and **p<0.001 versus placebo. Abbreviations: LSM=least squares mean; PG=plasma glucose; SE=standard error.
